# Burden of Illness in People with Alzheimer’s Disease: A Systematic Review of Epidemiology, Comorbidities and Mortality

**DOI:** 10.14283/jpad.2023.61

**Published:** 2023-06-01

**Authors:** Krista L. Lanctôt, J. Hviid Hahn-Pedersen, C. S. Eichinger, C. Freeman, A. Clark, L. R. S. Tarazona, J. Cummings

**Affiliations:** 1 Hurvitz Brain Sciences Program, Sunnybrook Research Institute; and Department of Psychiatry, University of Toronto, Toronto, Ontario Canada; 2 Novo Nordisk A/S, Søborg, Denmark; 3 Oxford PharmaGenesis, Oxford, UK; 4 Chambers-Grundy Center for Transformative Neuroscience, Department of Brain Health, School of Integrated Health Sciences, University of Nevada, Las Vegas, NV USA

**Keywords:** Alzheimer’s disease, prevalence, comorbidities, morbidity, mortality

## Abstract

**Background:**

Alzheimer’s disease (AD) is the most common neurodegenerative disease worldwide, and an updated quantification of its impact on morbidity, disability, and mortality is warranted. We conducted a systematic literature review, focusing on the past decade, to characterize AD and assess its impact on affected individuals.

**Methods:**

Searches of Embase, MEDLINE, and the Cochrane Library were conducted on August 7, 2020 and updated on November 10, 2021. Observational studies from any country reporting incidence, prevalence, comorbidities, and/or outcomes related to disability and mortality/life expectancy, in people with mild cognitive impairment (MCI) due to AD, or mild, moderate, or severe AD dementia, were considered relevant.

**Results:**

Data were extracted from 88 studies (46 incidence/prevalence; 44 comorbidities; 25 mortality-/disability-related outcomes), mostly from Europe, the USA, and Asia. AD dementia diagnosis was confirmed using biomarkers in only 6 studies. Estimated 5-year mortality in AD was 35%, and comorbidity prevalence estimates varied widely (hypertension: 30.2–73.9%; diabetes: 6.0–24.3%; stroke: 2.7–13.7%). Overall, people with AD dementia were more likely to have cardiovascular disease or diabetes than controls, and 5-year mortality in people with AD dementia was double that in the age- and year-matched general population (115.0 vs 60.6 per 1,000 person-years).

**Conclusions:**

AD is associated with excess morbidity and mortality. Future longitudinal studies of population aging, incorporating biomarker assessment to confirm AD diagnoses, are needed to better characterize the course of MCI due to AD and AD dementia.

**Electronic Supplementary Material:**

Supplementary material is available in the online version of this article at 10.14283/jpad.2023.61.

## Introduction

**D**ementia affects an estimated 57 million people worldwide (1), and Alzheimer’s disease (AD) is the most frequent cause of dementia (2). AD dementia is characterized by specific changes in the brain, notably the deposition of beta-amyloid protein as extracellular plaques and the presence of neurofibrillary tangles composed of phosphorylated tau protein (3). AD also has a preclinical phase and prodromal manifestations that are usually not taken into account in prevalence estimates.

Deficits in memory, language, and problem-solving may initially present as mild cognitive impairment (MCI) (4), which is not severe enough to affect functioning, whereas dementia is characterized by progressive cognitive deterioration and limitations to functioning (5) and is ultimately fatal. Nearly all individuals with MCI due to AD progress eventually to AD dementia if observed for long enough (6); in a cohort of 18,000 US individuals, the annual probability of progression to mild AD dementia at the age of 65 years for those with MCI due to AD was estimated to be 21% (7). Research diagnostic criteria for MCI due to AD and AD dementia incorporate assessment of biomarkers, alongside clinical evaluations and neuropsychological testing (8); these guidelines do not currently recommend the use of biomarkers to diagnose AD in clinical practice (6, 9). Although the vast majority of treated patients with AD receive therapies only for symptoms, two treatments have been approved by the US Food and Drug Administration for MCI due to AD and mild AD dementia (10, 11). As these therapies target beta-amyloid (10, 11), an increase in biomarker-defined AD is anticipated for the future.

The total number of deaths attributable to AD dementia has increased during the past 20 years, making it one of the leading causes of death in the USA (12). AD dementia is usually diagnosed in people in their mid-sixties or older; consequently, people with AD dementia commonly have comorbidities, such as cardiovascular disease (CVD). There is evidence that risk factors common to comorbidities and AD, such as chronic inflammation, can mean that individuals with comorbidities are at an increased risk of developing AD (13); however, the interplay between comorbidities, development and progression of AD dementia, and patient outcomes remains incompletely understood (14). Many large burden of disease studies, such as the Global Burden of Disease studies (15), group AD dementia with other dementias under the overarching term ‘dementia’, and as such, there is little information available to assess the relationship between comorbidity burden, disability, and mortality in individuals with AD specifically.

AD remains a key public health priority worldwide, and identification and synthesis of recent data are required to quantify its true impact. We designed and conducted a systematic literature review (SLR) to identify current evidence on the prevalence and incidence of AD and its effects on mortality and life expectancy, and to assess the relationships between AD, comorbidities, and disability.

## Materials and Methods

### Systematic literature review

The SLR was designed to identify relevant data on the burden of AD from observational studies of people with MCI due to AD, or mild, moderate, or severe AD dementia. Outcomes of interest were incidence and prevalence, comorbidities, mortality, life expectancy, and disability. To identify evidence on the latter two outcomes, search terms were included for years of life lost (YLLs), a measure of premature mortality, years lived with disability (YLDs), and disability-adjusted life-years (DALYs), which express the impact of disability in terms of how many years of healthy life have been lost. The study protocol was designed and conducted in line with the 2009 Preferred Reporting Items for Systematic Reviews and Meta-Analyses (PRISMA) guidelines (16), and registered with PROSPERO (registration number: CRD42022297125).

Searches were conducted on August 7, 2020, and updated on November 10, 2021 to capture literature published since the original searches; Table S1 and Table S2 show the search strings. Titles and abstracts were screened by one researcher to determine whether they met the eligibility criteria (Table 1). For inclusion, studies were required to specify which severity or stages of AD were represented in the population; all staging methods and criteria were considered valid. Journal articles published from 2010 onwards and conference abstracts published from 2015 onwards were considered relevant in screening. Primary publications were included, but review articles were not; reference lists from systematic reviews and meta-analyses were cross-checked for relevant articles. Only English language publications were included. There was no restriction by study geography. All publications meeting the criteria were obtained as full articles and reassessed against the eligibility criteria.

**Table 1:** Eligibility criteria for the systematic literature review

Populations	Patients with Alzheimer’s disease^a^ (from mild cognitive impairment to severe dementia of the Alzheimer’s type); this includes: (1) MCI of the Alzheimer’s type; (2) mild dementia of the Alzheimer’s type; (3) moderate dementia of the Alzheimer’s type; (4) severe dementia of the Alzheimer’s type
Interventions	Any or none
Comparators	Any or none
Outcomes	Burden of disease outcomes encompassing: incidence; prevalence; comorbidities; mortality; life expectancy; disability-adjusted life years (DALYs); years of life lost (YLLs); years lived with disability (YLDs)
Study design	• Observational/RWE studies will be included • Animal / *in vitro* studies, case studies and reports, reviews and editorials will be excluded
Date restrictions	• 2010–present for all studies, except conference abstracts • 2015–present for all conference abstracts
Language restrictions	English language
Publication type	All primary publications and systematic literature reviews^b^
Country	Not restricted

a. For inclusion, studies were required to specify stage/severity. Inclusion was not limited by staging system used. b. Data were not extracted from systematic literature review articles; however, the reference lists were cross-checked for any relevant sources. MCI, mild cognitive impairment; RWE, real-world evidence.

### Data extraction and prioritization

Data were extracted from studies published 2015–present, to identify the most recent and relevant data. For prevalence and incidence, data from studies that selected populations for the presence of dementia or neurological conditions were not extracted, because those data would not provide an accurate estimate of AD prevalence or incidence in the general population. For comorbidities, data were extracted from publications that also reported epidemiology or mortality data and, to assess the potential impact of AD on comorbidity prevalence, from any publications that reported comorbidities for both individuals with AD and controls. Detailed data, including study setting and methods, patient characteristics, and study results, were entered into a data extraction table and quality checked by an independent reviewer.

In this manuscript, only AD-specific data are reported, and data from populations that included individuals with non-AD dementias, only some of whom had AD, were not considered relevant. It should be noted, however, that because most AD diagnoses were not confirmed using biomarkers, some people diagnosed with AD dementia in the included studies are likely to have other types of dementia, or mixed dementia.

In this manuscript, we have used data from relevant studies to address two key aims. First, we characterize AD, collating data on incidence and prevalence, comorbidities in people with AD, mortality, and survival. Second, we aimed to understand the impact of AD on comorbidities and mortality by assessing studies comparing data between individuals with and without AD. One study that assessed the disability associated with AD as part of the wider impact of dementia is also discussed in the manuscript.

## Results

### Search results

**Figure 1:**
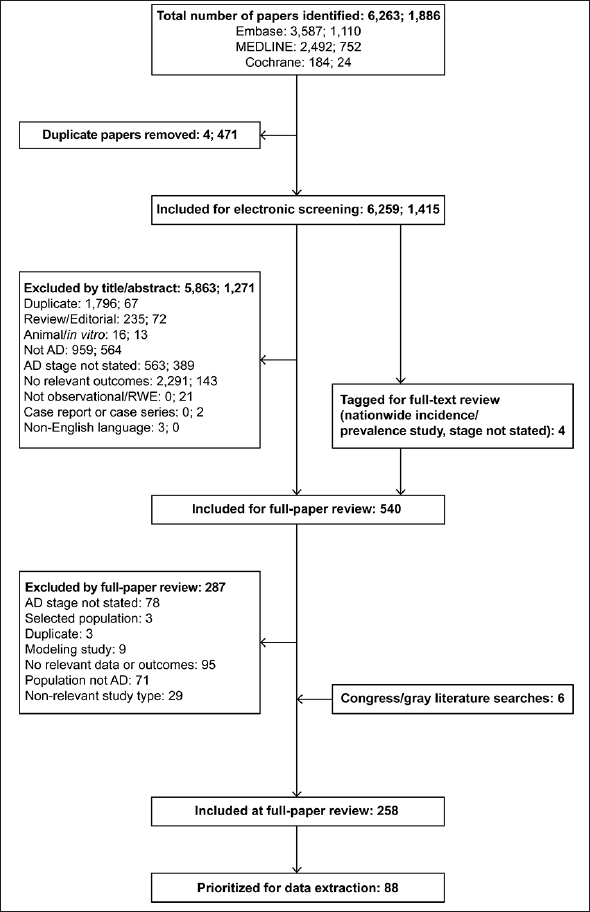
PRISMA diagram for the SLR The two sets of numbers at the search and screening stages indicate the numbers of references identified in the original SLR conducted in August 2020 and the update in November 2021, respectively. From full-paper review onwards, duplicates across the two searches had been identified and removed. AD, Alzheimer’s disease; RWE, real-world evidence; SLR, systematic literature review.

In total, 6,259 papers identified in the original SLR and 1,415 papers identified in the SLR update were included for screening by abstract and title, resulting in 540 references included for full-paper review (see PRISMA flow diagram in Figure 1). Cross-checks with a separate targeted literature review on AD resulted in the inclusion of three additional references, and three references were included from congress searches.

In total, 258 references met the inclusion criteria for full-paper review, and data were extracted from 88 studies. Some studies reported more than one type of data; Figure S1A shows the overlap between categories. Overall, 46 studies reported epidemiology data (17–62), 44 studies reported data on comorbidities (17, 19, 25, 27, 28, 34, 36, 37, 39, 42, 44, 60, 63–94), and 25 studies reported data on mortality, survival, and disability (30, 36, 42, 44, 60, 81, 82, 84, 85, 87, 88, 90–92, 94–104).

### Data sources and study designs

In total, 36 studies reported data from Europe (20, 22–24, 28, 30, 33, 35, 37, 48, 52, 55–58, 60, 63, 65, 73–77, 82, 86–88, 90–93, 95, 96, 98, 101, 104) and 22 studies reported data from Asia (25, 31, 38, 39, 43, 59, 62, 66, 68–72, 78–81, 83, 85, 89, 94, 99). Seventeen studies reported data from the USA (17–19, 26, 34, 36, 40–42, 45, 49–51, 61, 100, 102, 103). Two studies reported data from each of Australia (27, 44), North Africa (32, 46), Brazil (54, 64), and one study reported data from Cuba (67). Four studies reported data from multiple countries (47, 53, 84, 97), and two did not report country (21, 29). The countries represented in the data identified are shown in Figure S1B. Approximately half of the studies identified used primary data collection and a similar number were retrospective studies using secondary data. Most data were from dementia-based cohorts, national registries, and electronic medical records; however, in some publications individuals were recruited directly from hospitals or primary care settings.

### AD diagnosis and staging

In most studies, AD dementia was diagnosed using neurological and neuropsychiatric assessments, and in some cases brain imaging and laboratory tests. Most frequently, diagnoses were made using the National Institute of Neurological and Communicative Disorders and Stroke–Alzheimer’s Disease and Related Disorders Association (NINCDS-ADRDA) criteria, the Diagnostic and Statistical Manual of Mental Disorders (DSM)-IV criteria, or the criteria proposed by the National Institute on Aging–Alzheimer’s Association (NIA-AA) workgroup. A minority of publications reported any AD biomarker data for included individuals and only six studies used biomarkers to confirm the diagnosis of AD dementia (23, 30, 33, 38, 73, 92). Four of these studies assessed both amyloid and tau levels in cerebrospinal fluid (CSF) (total tau and p-tau in three (30, 73, 92); p-tau in one (33)), of which one also conducted brain magnetic resonance imaging (MRI) (92). Honda et al. (2019) reported the results of an autopsy series assessing tau pathology over 31 years in Japan (38), and the remaining publication (Bouteloup et al., 2019) carried out MRI and amyloid positron emission tomography (PET) scans on a sub-sample of individuals, but did not assess tau or p-tau levels (23).

### Characterization of AD

#### Prevalence of MCI due to AD and AD dementia

Estimates of AD prevalence varied widely across studies, owing to differences in study settings and the ages of the populations being assessed, among other factors (Table S2). Six studies reported single estimates of AD prevalence in the general older population or in population-based cohorts (32, 37, 39, 49, 52, 56). Only one of these studies (Vlachos et al., 2020) reported the prevalence of MCI due to AD, which was found to be 8.4% in a cohort of older (≥ 65 years) people in Greece (56). The prevalence of AD dementia was reported in four studies (32, 37, 39, 49), and ranged from 0.8% in Italy (Grande et al., 2020) (37) to 14.5% in the USA (Rajan et al., 2019) (49). The sixth study (Ruano et al., 2019) reported the combined prevalence of MCI due to AD, and AD dementia, in Portugal, which together was 1.8% (1.4% MCI due to AD and 0.4% AD) (52). Three studies reported changes in AD prevalence over time; however, the settings, study periods and patient characteristics, such as age, in these analyses varied (Table S2).

#### Incidence of MCI due to AD and AD dementia

Six studies reported estimates of AD incidence in the general population (Table S2) (20, 40, 49, 57, 58, 62). Kirson et al. (2020) estimated the incidence of AD dementia in a random sample of US Medicare beneficiaries using International Classification of Diseases codes. Incidence decreased from 1.5% in 2007 to 1.1% in 2014 (40). Rajan et al. (2019) reported an incidence of 2.3%, based upon data from individuals ≥ 65 years old in the Chicago Health and Aging Project, a population-based study standardized to the US population (49). Yuan et al. (2016) reported that the crude incidence of AD dementia per 1,000 person-years was 4.9 in 16,921 individuals aged 55 years or older in China (62), and Andreu-Reinon et al. (2020) reported an age-adjusted incidence of AD dementia of 5.5 per 1,000 person-years among people ≥ 65 years old in a Spanish population, standardized to the 2013 European Standard Population (20). The highest estimates were reported in two analyses from the Hellenic Longitudinal Investigation of Aging and Diet study in Greece, which included individuals aged 65 years old or over. The incidence of MCI due to AD per 1,000 person-years was 34.1 (Vlachos et al., 2021a) (57), and the incidence of AD dementia was 16.3 (Vlachos et al., 2021b) (58).

#### AD dementia prevalence by stage

Four studies reported estimates of the prevalence of AD dementia by stage at diagnosis or referral (Table 2); only one included MCI in these assessments (Yuan et al., 2021) (61). Two were cross-sectional studies of individuals referred to outpatient clinics. Souza et al. (2019) found that, in a cohort of 256 individuals in Brazil, most presented with mild AD dementia (54). In the second study, El Tallawy et al. (2019) reported data from 126 people in Egypt invited for an assessment due to suspected dementia. Similar proportions had mild and moderate AD dementia, and the remainder had severe AD dementia (32). An analysis by Yuan et al. (2021) of individuals in the population-based US Framingham Heart Study found that mild AD dementia was the most prevalent stage of AD dementia at diagnosis (50.4% of 607 individuals with AD dementia). In the full population (N = 1192), which included individuals with MCI and AD dementia, 49.1% had MCI (29.5% MCI that did not progress to AD; 19.5% MCI that progressed to AD), 25.7% had mild AD dementia, 15.4% had moderate AD dementia, and 9.8% had severe AD dementia. (61). In a study by Chua et al. (2019) assessing data from people who presented with AD dementia at a geriatric medicine memory clinic in Singapore between 2005 and 2017 (25), the proportion presenting with mild AD dementia increased whereas the proportion presenting with moderate AD dementia decreased over time (Figure S2).

**Table 2:** Studies reporting the prevalence of AD dementia stages

**First author and year**	**Country**	**Setting**	**N with AD**	**Mild AD dementia**		**Moderate AD dementia**		**Severe AD dementia**	
				**Definition**	**Prevalence (%)**	**Definition**	**Prevalence (%)**	**Definition**	**Prevalence (%)**
Souza et al. (2019) (56)	Brazil	Individuals referred to a memory and behavior disorder outpatient clinic	256	CDR score 1	46.5	CDR score 2	37.1	CDR score 3	16.4
El Tallawy et al. (2019) (34)	Egypt	Community-dwelling individuals; those with suspected dementia were invited to hospital for assessment	126	MMSE score 17–21	41.3	MMSE score 9–16	43.7	MMSE score < 9	15.0
Yuan et al. (2021) (63)	USA	Population-based Framingham Heart Study	607	Clinical judgment	50.4	Clinical judgment	30.3	Clinical judgment	19.3
Chua et al. (2019) (27)	Singapore	Geriatric medicine memory clinic	2005: 46 2017: 72	FAST score 4	2005: 41.3 2017: 68.1	FAST score 5	2005: 45.7 2017: 27.8	FAST score 6–7	2005: 13.0 2017: 4.2

AD, Alzheimer’s disease; CDR, Clinical Dementia Rating; FAST, Functional Assessment Staging Tool; MMSE, Mini-Mental State Examination.

#### Prevalence of comorbidities in AD

**Figure 2:**
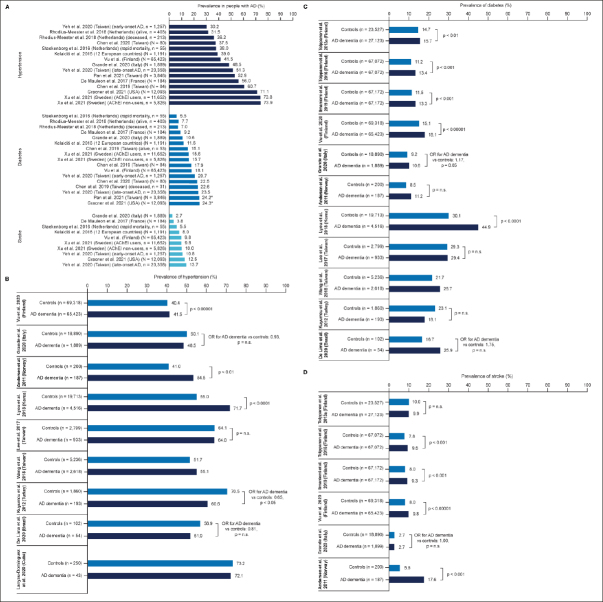
Prevalence of selected comorbidities in people with AD (A), and prevalence of hypertension (B), diabetes (C) and stroke (D) in people with AD and control individuals Further details: Asterisk (*) denotes studies that specify T2D. For panel (A), studies are presented in order of increasing prevalence. For panel (B), studies are presented by region. Panel (A): Chen et al. 2019: study period 2012–2016. ‘Alive’ refers to individuals newly diagnosed with probable AD who were alive at the end of the study period (age [mean ± SD], alive: 85.8 ± 3.1 years; deceased: 87.9 ± 4.7 years) (81). Chen et al. 2020: study period 2015–2016 (80). de Mauleon et al 2017: study period 2007–2011; ‘stroke’ is reported as history of or ongoing ischemic and hemorrhagic stroke (28). Gracner et al. 2021: study period 2011–2013 (36). Grande et al. 2020: study period 2002–2006; hypertension, diabetes and stroke are reported for the ≥ 10-year medical history (37). Kelaiditi et al. 2016: study period 2003–2005; the 12 European countries are not specified (84). Pan et al. 2021: study period 2001–2019 (89). Rhodius-Meester et al. 2018: study period 2000–2014; alive refers to individuals alive after mean ± SD follow-up of 4.9 ± 2.0 years; hypertension and diabetes are reported as history of hypertension and/or use of antihypertensive drugs, and history of diabetes mellitus and/or use of antidiabetic drugs, respectively (90). Staekenborg et al. 2016: study period 2000–2013; rapid mortality is defined as death within ≤ 2 years after diagnosis; stroke includes TIA (92). The prevalence of hypertension is reported as 38% in the publication, but the number of cases is reported as 20 of 55 individuals. Vu et al. 2020: study period 2005–2011; comorbidities are reported as prevalence at the time of AD diagnosis (93). Xu et al. 2021: study period 2007–2017 (60). Yeh et al. 2020: Study period 2000–2012. mean ± SD age for early-onset AD group: 61 ± 4; late-onset AD group: 78 ± 6 (94). Panels (B–D): Andersen et al. 2011: study period 2006–2008 (63). de Lima et al. 2020: study period NR (64). Grande et al. 2020: study period 2002–2006 (37). Ilmaniemi et al. 2019: study period 2005–2015 (65). Kuyumcu et al. 2012: study period NR (66). Lanyau-Dominguez et al. 2020: study period NR (67). Lee et al. 2017: study period 2000–2010 (69). Lyou et al. 2018: study period 2004–2013 (72). Tolppanen et al. 2013a: study period 2002–2009; ‘stroke’ is reported as history of stroke (74). Tolppanen et al. 2016: study period 2005–2012 (77). Vu et al. 2020: study period 2005–2011 (93). Wang et al. 2018: study period 2001–2011 (78). AChEI, acetylcholinesterase inhibitor; AD, Alzheimer’s disease; NR, not reported; OR, odds ratio; RWE, real-world evidence; SLR, systematic literature review; T2D, type 2 diabetes; TIA, transient ischemic attack.

Thirteen studies (Figure 2A and Table S4) reported data on hypertension, diabetes (type 1 or 2), or CVD in patient cohorts with MCI due to AD or AD dementia, including one study (Xu et al., 2021) (60) that comprised people with AD dementia or mixed AD dementia (28, 36, 37, 60, 80, 81, 84, 88–90, 92–94). These data were typically included in summary tables of baseline characteristics, and the development of comorbidities relative to the timing of AD dementia diagnosis was not clear in most publications. The prevalence of hypertension ranged between 30.2% (Yeh et al., 2020, in people with early-onset AD) (94) and 73.8%/73.9% (Xu et al., 2021, in acetylcholinesterase inhibitor users and non-users, respectively) (60). The prevalence of diabetes ranged between 6.0% (Staekenborg et al., 2016, in people with AD dementia [diagnosis supported by biomarker assessment] with rapid mortality) (92) and 24.3% (Gracner et al., 2021, type 2 diabetes only) (36). The prevalence of stroke ranged between 2.7% (Grande et al., 2020) (37) and 13.7% (Yeh et al., 2020, in people with late-onset AD) (94). Data on the prevalence of various other cardiovascular conditions and risk factors were also reported, including, heart failure, myocardial infarction, and dyslipidemia (Table S4).

#### Mortality in AD dementia

Six studies reported estimates of all-cause mortality in AD dementia (Table 3) (30, 44, 90, 94, 98, 104). Two additional studies reported data on life expectancy only (44, 81) (not presented). Mortality estimates could not be readily compared across all studies owing to differences in follow-up periods; however, three studies had a similar duration of follow-up. Mank et al. (2021) used data from the Amsterdam Dementia Cohort (98), and Rhodius-Meester et al. (2018) conducted a prospective study using the same cohort (90), meaning that the populations in the two studies were similar in terms of age (Mank et al., 2021: mean ± standard deviation [SD]: 65 ± 7 years; Rhodius-Meester et al., 2018: 66 ± 7 years [alive at end of follow-up] and 69 ± 9 years [deceased at end of follow-up]); all had AD dementia. Degerman Gunnarsson et al. (2016) used data from a memory clinic in Sweden. Included individuals had a median age of 70 years (range: 46–86) and 57% had MCI due to AD rather than AD dementia (30); AD diagnosis was supported by assessment of CSF total tau levels. In each of these three analyses, 35% of people died over approximately 5 years of follow-up.

**Table 3:** Mortality among people with MCI due to AD or AD dementia

**First author and year**	**Country (study period)**	**Data source and design**	**Population**	**Age, years**	**Follow-up, years**	**Mortality**
Vazquez Justes et al. (2021) (106)	Spain (NR)	Memory clinic (Prospective)	N = 98 with AD dementia^a^	> 60 (inclusion criterion)	Mean:^b^ 3	11 people (11%) died
Mank et al. (2021) (100)	Netherlands (NR)	Amsterdam Dementia Cohort (Retrospective)	N = 1,179 with AD dementia^a^	Mean ± SD: 65 ± 7	Mean ± SD: 4.5 ± 2.7	413 people (35%) died
Rhodius-Meester et al. (2018) (92)	Netherlands (2000–2014)	Amsterdam Dementia Cohort (Prospective)	N = 616 with AD dementia^c^ (403 alive at end of follow-up, 213 deceased)	Mean ± SD Alive: 66 ± 7 Died: 69 ± 9	Mean ± SD: 4.9 ± 2.0	35% of people died
Degerman Gunnarsson et al. (2016) (32)	Sweden (2003–2011)	Memory Clinic, Upsala University Hospital (Retrospective)	N = 234 (134 with MCI due to AD; 100 with mild-to-moderate AD dementia)^d^	Median (range): 70 (46–86)	Median (range): 4.9 (2–11)	35% of people died
Yeh et al. (2020) (96)	Taiwan (2000–2012)	NHIRD; National Mortality Registry (Retrospective)	n = 1,257 with EOAD^e^	Mean ± SE EOAD: 60.6 ± 4.4	Mean ± SE EOAD: 8.2 ± 0.1	139 people with EOAD (11%) died
			n = 20,358 with LOAD^e^	Mean ± SE LOAD: 77.5 ± 6.4	Mean ± SE LOAD: 6.8 ± 0.0	5,554 people with LOAD (27%) died
Loi et al. (2020) (46)	Australia (1992–2014)	Neuropsychiatry inpatients (Retrospective)	N = 115 with AD dementia^a^	Mean ± SD 59.9 ± 11.1 (at AD onset)	NR (assumed up to 22 years)	89 people (77%) died

a. Method of diagnosis NR. b. Assumed to be mean, but NR. c. Baseline MMSE score ≥ 16. d. Diagnosed using NINCDS-ADRDA and DSM-IV criteria. CT and/or MRI, lumbar puncture, and cognitive assessments. Individuals with neuroimaging suggesting a mixture of AD and cerebrovascular pathology were not included. e. AChEIs prescription plus MMSE score 10–26 or CDR score 1–2. CT and/or MRI. EOAD was defined as age of onset between 40 and 64 years. LOAD was defined as age of onset ≥ 65 years. AChEI, acetylcholinesterase inhibitor; AD, Alzheimer’s disease. CDR, Clinical Dementia Rating. CT, computed tomography. DSM-IV, Diagnostic and Statistical Manual of Mental Disorders (Fourth Edition). EOAD, early-onset AD dementia. LOAD, late-onset AD dementia. MCI, mild cognitive impairment. MMSE, Mini-Mental State Examination. MRI, magnetic resonance imaging; NHIRD, National Health Insurance Research Database. NINCDS-ADRDA, National Institute of Neurological and Communicative Disorders and Stroke–Alzheimer’s Disease and Related Disorder Association. NR, not reported.

### Burden of AD

#### Comorbidities

Nineteen studies compared the prevalence of comorbidities in people with AD dementia with those in the general population or in cognitively healthy control individuals (37, 63–79, 93) (Table S5).

Nine studies reported data on the prevalence of hypertension in people with AD dementia, compared with control individuals (Figure 2B) (37, 63, 64, 66, 67, 69, 72, 78, 93). The prevalence of hypertension varied across studies but was generally similar in people with AD dementia and controls. Lyou et al. (2018) (72) found a higher prevalence in the cohort with AD dementia (71.7% vs 55.0% in controls), but Kuyumcu et al. (2012) (66) found a lower prevalence (60.6% vs 70.5% in controls).

Eleven studies reported data on the prevalence of diabetes in AD dementia and controls (Figure 2C) (37, 63–66, 69, 72, 74, 77, 78, 93). In most studies, diabetes was slightly more common in individuals with AD dementia, or prevalence was similar between the groups. The largest difference between AD dementia and controls reported was in Lyou et al. (2018) (72) (AD dementia, 44.9%; controls, 30.1%; p < 0.0001). Of the eleven studies, only Kuyumcu et al. (2012) (66) found a lower prevalence of diabetes in AD dementia than in controls (18.1% vs 23.1%).

Eight studies reported data on stroke (Figure 2D) (37, 63, 65, 71, 74, 76, 77, 93). In two studies, the prevalence of stroke was similar in people with AD dementia and in controls (37, 74), in three studies stroke was slightly more common in people with AD dementia (p < 0.001 in all three studies) (65, 77, 93), and in one study, stroke was considerably more common in people with AD dementia than in controls (17.6% vs 5.5%), but this difference was not statistically significant in a model adjusted for age and sex (63). Two studies reported hazard ratios rather than prevalence. Lee et al. (2019b) (71) found that people with AD dementia were at greater risk of stroke than controls (hazard ratio: 2.87 [95% confidence interval: 2.71–3.04]). Tolppanen et al. (2013c) found that hemorrhagic stroke was slightly more common in AD dementia than in controls, but ischaemic stroke was less common (Table S5) (76). In five studies, the prevalence of CVD or specific cardiovascular conditions other than stroke was higher in AD dementia cohorts than in controls (63, 66, 77, 78, 93); however, Grande et al. (2020) (37) found similar or slightly lower prevalence of cardiovascular conditions in AD dementia than in controls (Table S5).

#### Disability and mortality

Only one of the identified publications assessed the impact of AD dementia on disability. Moon et al. (2021) examined a cohort of approximately 6,500 South Korean people aged 65 years or older, and found that AD dementia accounted for the greatest proportion of total DALYs (33%) and YLDs (37%) attributed to all-cause MCI and all-cause dementia in this population (99).

Similarly, only one publication compared mortality between people with AD dementia and the general population. Xu et al. (2021) used Swedish Dementia Registry data to compare mortality in 10,129 individuals with AD dementia (mean age: 81 years) against age- and year-matched controls from the general Swedish population. Over 5 years, the death rate of people with AD dementia was 115.0 per 1,000 person-years, which was approximately double that of the controls (60.6 per 1,000 person-years) (60).

## Discussion

This SLR was designed to characterize AD and to estimate the burden that it imposes on affected individuals. We found evidence to indicate that people with AD dementia are more likely to have CVD, including stroke, than people without AD; however, no clear pattern was apparent for hypertension. In addition, several studies indicated that diabetes is relatively more common in people with AD dementia. There is evidence that people with AD dementia have higher mortality than those without. We have identified some key gaps in the available evidence, which can be used to inform further research.

The causes of the observed relationships between comorbidities and the development or progression of AD dementia, such as shared risk factors or disease mechanisms, warrant further investigation. Published evidence implies multifacted associations between comorbidities, such as CVD and type 2 diabetes, and the development of cognitive impairment (105, 106), and more detailed longitudinal studies that track the timing of comorbidity development relative to the development and progression of AD are needed to elucidate this complex relationship further.

Only one study that reported evidence on disability in AD dementia was identified, and it did not compare AD dementia with the general population (Moon et al. 2021) (99). Although several large Global Burden of Disease analyses have shown that dementia is a major contributor to mortality and disability in older patient populations in the USA, Europe and worldwide (15, 107–109), the grouping of AD with other dementias means that its specific impact on these factors remains less well characterized. This is reflected in a general trend identified in our SLR: although we aimed to focus on studies reporting data for AD dementia or MCI due to AD, a number of the studies grouped AD with either all-cause MCI or non-AD types of dementia, and therefore not all could be used to characterize AD dementia specifically.

The results of our SLR highlight how variability across studies leads to difficulties in collating relevant data to characterize AD dementia in specific countries and on a global scale. Disparities across studies in terms of patient age, study setting, ascertainment method and staging of AD dementia, among other factors, contributed to wide variation in the results. Importantly there were disparities in the method of AD diagnosis across studies. Some studies identified people with AD dementia in retrospective data using diagnosis codes rather than clinical assessment, and most studies did not confirm AD dementia diagnoses via assessment of amyloid and tau. Although AD biomarkers are recommended as a diagnostic tool predominantly in research settings rather than in clinical practice (6, 110), there is a large and growing body of evidence indicating that biomarker assessments can influence AD dementia diagnoses in clinical contexts. It is estimated that 10–30% of individuals diagnosed with AD by experts do not show neuropathological changes consistent with AD at autopsy, in PET scans or CSF assessments (6). Use of information derived from amyloid PET scans has been shown to influence subsequent decision making in clinical management of individuals with dementia of uncertain etiology, suggesting that it has practical benefits in reaching a diagnosis (111). It is likely that some people considered to have AD dementia in the studies identified in this SLR were misdiagnosed or had other concomitant diagnoses, which should be taken into account when interpreting the results.

It is probable that many of the prevalence and incidence data identified in this SLR underestimate the true number of individuals affected by AD, in part because few examined MCI due to AD specifically (as opposed to all-cause MCI) or confirmed AD pathology using biomarkers. A recent SLR and modeling study combined data from meta-analyses estimating the prevalence of MCI, AD dementia, and beta-amyloid positivity to calculate global estimates for biomarker-positive AD (112). Overall, it was estimated that 22% of people worldwide aged 50 years or older have AD dementia (1.7%), MCI due to AD (termed ‘prodromal AD’; 3.7%) or preclinical AD (defined as beta-amyloid positivity in people with normal cognition or subjective cognitive impairment; 17%) (112). Although the estimate for AD dementia prevalence falls within the range identified in our SLR, the larger estimate when preclinical AD and MCI due to AD are taken into account highlights the potential burden of AD that is not captured by studies including only individuals with a formal diagnosis of AD dementia.

The previously published modeling study indicated that estimation of the worldwide impact of AD is compromised by the paucity of data in some geographical regions. This is supported by the findings of our SLR. European and Asian data were drawn only from subsets of countries, and North American data were only from the USA. Data from South America, Africa, the Middle East, and Oceania were few or absent.

A requirement for mention or specification of AD stage or severity in the publication title or abstract was included in the SLR design to maintain focus on AD, and to identify evidence on the impact of AD progression. To identify the most recent evidence, data extraction was restricted to publications from 2015 onwards. Although a wide range of outcomes were included, it is likely that some relevant evidence was not identified in searches owing to the absence of key terms in the title or abstract. Comorbidities were examined as a broad outcome, and search terms for specific comorbidities were not included. Future, focused reviews examining specific comorbidities of interest would be highly valuable, and new studies and literature reviews should also capture the impact of the COVID-19 pandemic on care and outcomes in AD. Research is needed to investigate the biological interplay between these two diseases, as well as the effect that the sociological changes imposed by the pandemic have had on diagnosis, treatment, and support for people affected by AD dementia.

In conclusion, we have found evidence that AD is associated with morbidity, including CVD, stroke, and diabetes, and mortality. Further research in older populations is needed to quantify these risks and to understand the factors underlying AD dementia development, progression, and outcomes. Particularly given the emergence of disease modifying therapies, future longitudinal studies of population aging will be vital to track the disease course and its association with other conditions. To provide maximum value to clinicians, patients, and researchers, such studies should differentiate AD dementia from other dementias by using best practice for diagnosis and staging, incorporating assessment of biomarkers along with clinical examination and cognitive testing.

### Electronic supplementary material

Burden of Illness in People with Alzheimer’s Disease: A Systematic Review of Epidemiology, Comorbidities and Mortality, approximately 488 KB.
