# Supplementary material for: Burden of Illness in People with Alzheimer’s Disease: A Systematic Review of Epidemiology, Comorbidities and Mortality
Source: J Prev Alzheimers Dis. 2023 Jun 1;11(1):97–107. doi: 10.14283/jpad.2023.61 (PMC10225771; doi:10.14283/jpad.2023.61)
Supplement: Supplementary file 1 — Burden of Illness in People with Alzheimer’s Disease: A Systematic Review of Epidemiology, Comorbidities and Mortality, approximately 488 KB. [file 42414_2023_61_MOESM1_ESM.docx]

Burden of Illness in People with Alzheimer’s Disease: A Systematic Review of Epidemiology, Comorbidities and Mortality

Krista L. Lanctôt^1^, Julie Hviid Hahn-Pedersen^2^, Christian Stefan Eichinger^3^, Caroline Freeman^3^, Alice Clark^2^, Luis Rafael Solís Tarazona^2^, Jeffrey Cummings^4^

^1^Hurvitz Brain Sciences Program, Sunnybrook Research Institute; and Department of Psychiatry, University of Toronto, Toronto, Ontario, Canada

^2^Novo Nordisk A/S, Søborg, Denmark.

^3^Oxford PharmaGenesis, Oxford, UK.

^4^Chambers-Grundy Center for Transformative Neuroscience, Department of Brain Health, School of Integrated Health Sciences, University of Nevada, Las Vegas, NV, USA

**Corresponding author:** Krista L. Lanctôt
Email address: [Krista.Lanctot@sunnybrook.ca](mailto:Krista.Lanctot@sunnybrook.ca)
Telephone: +1 416 480-6100; Ext: 2241

Abstract word count: 235/250

Main text word count (excluding legends and references): 4219

References: 112

Tables/figures: 3 tables; 2 figures

Supplementary Materials: 5 tables; 2 figures

Running title: Burden of Alzheimer’s disease

# Supplementary Materials

## Supplementary Methods

Systematic searches were performed in August 2020 and updated in November 2021, and were carried out using Embase, MEDLINE/Medline In-Process and the Cochrane Library via Ovid. The original SLR and the update searched for studies from two separate periods (January 2010–August 2020, and January 2020–November 2021, respectively), and included sufficient overlap between the search periods to allow for any delays in indexing published studies. Search terms (Table S1) were designed to detect studies reporting data on relevant outcomes in MCI due to AD or AD dementia. Abstracts from relevant congresses were reviewed: abstracts from the Alzheimer’s Association International Conferences in 2018–2021 were either covered in electronic searches or searched separately, and abstracts from the biennial International Conference on Alzheimer's and Parkinson's Diseases in 2021 were also searched. Gray literature searches were also carried out: relevant US guidelines, governmental organizations, clinical societies, patient advocacy sites, and central registry lists were searched via Google. Finally, the reference lists in a concurrent literature review to identify real-world data sources in AD were cross-checked for potentially relevant references.

Eligibility criteria for inclusion of studies reporting either prevalence or clinical data (Table 1) were developed according to the PICOS (patient, interventions, comparisons, outcomes, and study design) framework.

Titles and abstracts were screened in a single-blind manner to determine whether they met the eligibility criteria. All publications that met entry criteria for review were obtained as full articles and reassessed against the review criteria. Relevant data, along with information on study design and setting, patient demographics and disease status, were extracted from each included publication into a data extraction table.

## Figure S1. Distribution of studies across (A) outcomes of interest and (B) geographical regions


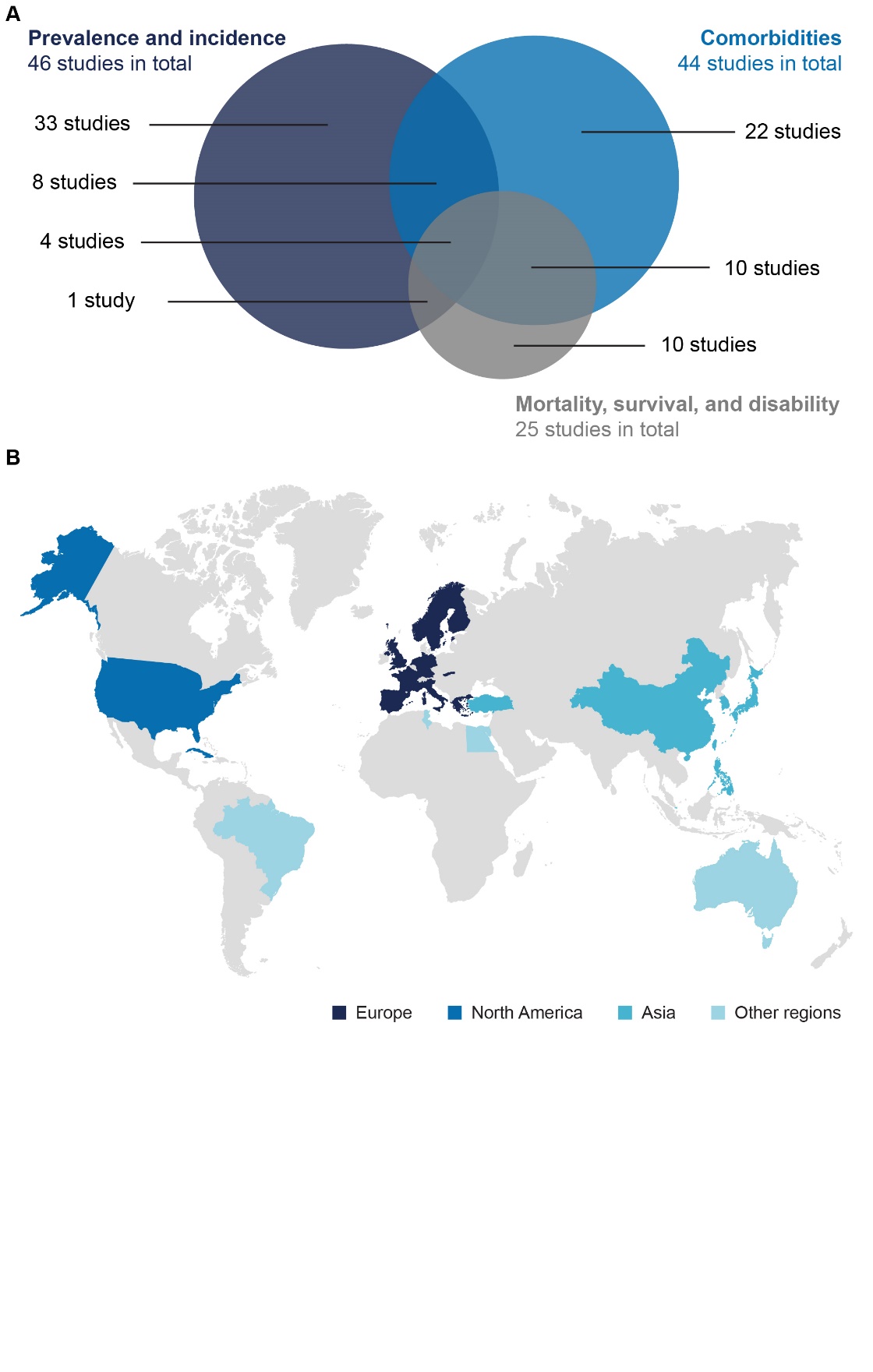


Panel A includes all studies in the SLR. Panel B includes all studies in which data were from a population in one country. Four studies reported data from multiple countries, and two studies did not report the country.

## Figure S2. Prevalence of AD stages over time in a geriatric medicine memory clinic in Singapore in Chua et al., 2019 ([27](#_ENREF_27))


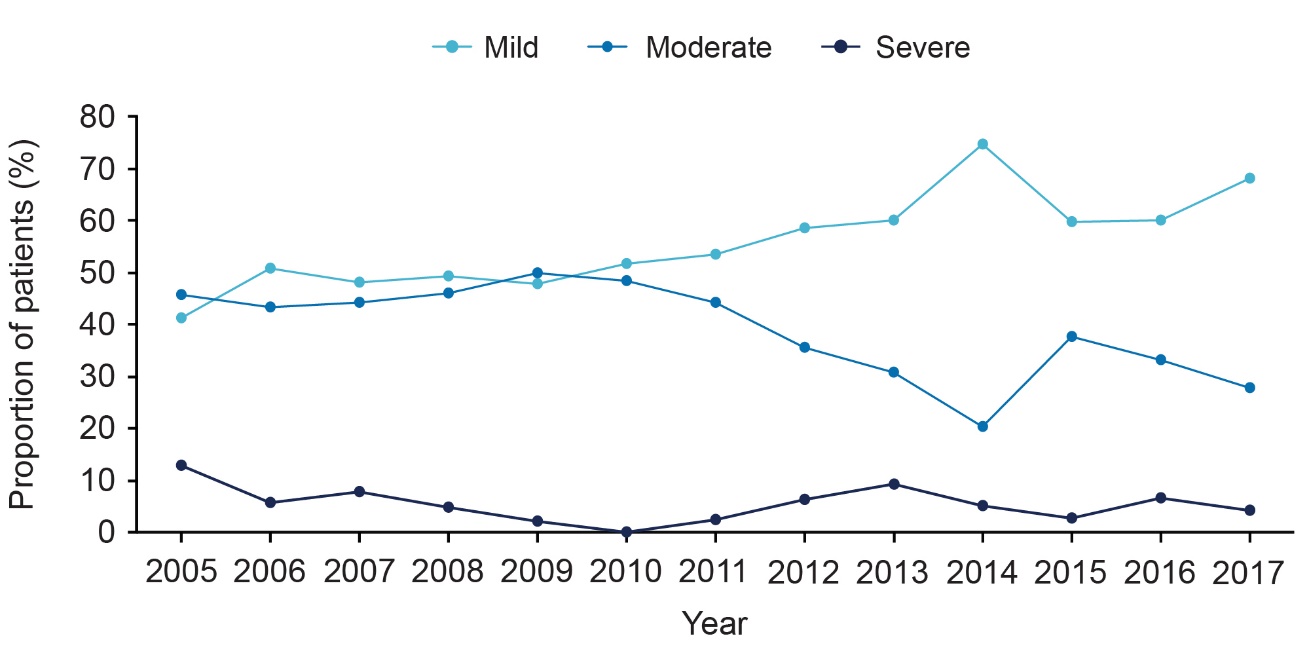


AD, Alzheimer’s disease.

## Table S1. Electronic search strings for the original SLR

| **Embase** | | |
| --- | --- | --- |
| **#** | **Searches** | **Results** |
| 1 | Alzheimer disease/ or Alzheimer*.ti,ab. | 240175 |
| 2 | epidemiology/ or epidemiology.ti,ab,kw. | 443735 |
| 3 | incidence/ or incidence.ti,ab,kw. | 1192638 |
| 4 | prevalence/ or prevalence.ti,ab,kw. | 1062509 |
| 5 | mortality/ or life expectancy/ or mortality rate/ | 845076 |
| 6 | (mortality or mortaliti*).tw,ot. | 1120144 |
| 7 | morbidity/ or comorbidity/ | 601977 |
| 8 | (morbidity or morbiditi* or comorbidity or comorbiditi*).tw,ot. | 787338 |
| 9 | (burden of disease or disability adjusted life year or disability-adjusted life year or DALY or years of life lost or YLL or years lived with disability or YLD).ti,ab,kw. | 18631 |
| 10 | or/2-9 | 3902711 |
| 11 | 1 and 10 | 22418 |
| 12 | Clinical study/ | 155587 |
| 13 | Case control study/ | 158995 |
| 14 | Family study/ | 26075 |
| 15 | Longitudinal study/ | 142491 |
| 16 | Retrospective study/ | 947119 |
| 17 | Prospective study/ | 618874 |
| 18 | Randomized controlled trials/ | 183816 |
| 19 | 17 not 18 | 612234 |
| 20 | Cohort analysis/ | 603593 |
| 21 | (Cohort adj (study or studies)).mp. | 311915 |
| 22 | (Case control adj (study or studies)).tw. | 135160 |
| 23 | (follow up adj (study or studies)).tw. | 63658 |
| 24 | (observational adj (study or studies)).tw. | 169968 |
| 25 | (cross sectional adj (study or studies)).tw. | 222528 |
| 26 | (registry or register$ or survey).ti,ab. | 1088282 |
| 27 | (real world or RWE).ti,ab. | 71771 |
| 28 | Real-life.ti,ab. | 33223 |
| 29 | or/12-16,19-28 | 3613132 |
| 30 | 11 and 29 | 5742 |
| 31 | (animal$ not human$).sh,hw. | 4385716 |
| 32 | 30 not 31 | 5698 |
| 33 | limit 31 to (editorial or erratum or letter or note or patent or reports or "conference review" or "review") | 145309 |
| 34 | 32 not 33 | 5698 |
| 35 | case study/ or case report.tw. | 488820 |
| 36 | 34 not 35 | 5673 |
| 37 | limit 36 to english language | 5476 |
| 38 | limit 37 to yr="2010 -Current" | 4159 |
| 39 | Conference Abstract.pt. | 3841901 |
| 40 | 38 and 39 | 1400 |
| 41 | limit 40 to yr=2015-current | 883 |
| 42 | 40 not 41 | 517 |
| 43 | 38 not 42 | 3642 |
| 44 | remove duplicates from 43 | 3587 |
| **MEDLINE** | | |
| # | Searches | Results |
| 1 | Alzheimer disease/ or Alzheimer*.ti,ab. | 157731 |
| 2 | epidemiology/ or epidemiology.ti,ab,kw. | 187951 |
| 3 | incidence/ or incidence.ti,ab,kw. | 857533 |
| 4 | prevalence/ or prevalence.ti,ab,kw. | 713135 |
| 5 | mortality/ or life expectancy/ or mortality rate/ | 58697 |
| 6 | (mortality or mortaliti*).tw,ot. | 764441 |
| 7 | morbidity/ or comorbidity/ | 137964 |
| 8 | (morbidity or morbiditi* or comorbidity or comorbiditi*).tw,ot. | 502528 |
| 9 | (burden of disease or disability adjusted life year or disability-adjusted life year or DALY or years of life lost or YLL or years lived with disability or YLD).ti,ab,kw. | 12953 |
| 10 | or/2-9 | 2473937 |
| 11 | 1 and 10 | 12048 |
| 12 | Epidemiologic studies/ or exp case control studies/ or exp cohort studies/ or Case control.tw. or (cohort adj (study or studies)).tw. or Cohort analy$.tw. or (Follow up adj (study or studies)).tw. or (observational adj (study or studies)).tw. or Longitudinal.tw. or Retrospective.tw. or Cross sectional.tw. or Cross-sectional studies/ or (registry or register$ or survey).ti,ab. or (real world or RWE).ti,ab. or Real-life.ti,ab. | 3609791 |
| 13 | 11 and 12 | 4645 |
| 14 | (animal$ not human$).sh,hw. | 4681337 |
| 15 | 13 not 14 | 4634 |
| 16 | limit 14 to (editorial or erratum or letter or note or patent or reports or "conference review" or "review") [Limit not valid in Ovid MEDLINE(R),Ovid MEDLINE(R) Daily Update,Ovid MEDLINE(R) In-Process,Ovid MEDLINE(R) Publisher; records were retained] | 197623 |
| 17 | 15 not 16 | 4634 |
| 18 | case study/ or case report.tw. | 2171363 |
| 19 | 17 not 18 | 4606 |
| 20 | limit 19 to english language | 4343 |
| 21 | limit 20 to yr="2010 -Current" | 2502 |
| 22 | remove duplicates from 21 | 2492 |
| **Cochrane** | | |
| # | Searches | Results |
| 1 | Alzheimer disease/ or Alzheimer*.ti,ab. | 11551 |
| 2 | epidemiology/ or epidemiology.ti,ab,kw. | 12745 |
| 3 | incidence/ or incidence.ti,ab,kw. | 117750 |
| 4 | prevalence/ or prevalence.ti,ab,kw. | 39822 |
| 5 | mortality/ or life expectancy/ or mortality rate/ | 1073 |
| 6 | (mortality or mortaliti*).tw,ot. | 87313 |
| 7 | morbidity/ or comorbidity/ | 4626 |
| 8 | (morbidity or morbiditi* or comorbidity or comorbiditi*).tw,ot. | 60903 |
| 9 | (burden of disease or disability adjusted life year or disability-adjusted life year or DALY or years of life lost or YLL or years lived with disability or YLD).ti,ab,kw. | 1146 |
| 10 | or/2-9 | 258294 |
| 11 | 1 and 10 | 1057 |
| 12 | Epidemiologic studies/ or exp case control studies/ or exp cohort studies/ or Case control.tw. or (cohort adj (study or studies)).tw. or Cohort analy$.tw. or (Follow up adj (study or studies)).tw. or (observational adj (study or studies)).tw. or Longitudinal.tw. or Retrospective.tw. or Cross sectional.tw. or Cross-sectional studies/ or (registry or register$ or survey).ti,ab. or (real world or RWE).ti,ab. or Real-life.ti,ab. | 301101 |
| 13 | 11 and 12 | 327 |
| 14 | (animal$ not human$).sh,hw. | 2101 |
| 15 | 13 not 14 | 327 |
| 16 | limit 14 to (editorial or erratum or letter or note or patent or reports or "conference review" or "review") [Limit not valid in ACP Journal Club,CDSR,CCTR,DARE,CLEED,CLHTA,CLCMR,CCA; records were retained] | 0 |
| 17 | 15 not 16 | 327 |
| 18 | case study/ or case report.tw. | 2234 |
| 19 | 17 not 18 | 326 |
| 20 | limit 19 to english language [Limit not valid in ACP Journal Club,CDSR,DARE,CLCMR,CCA; records were retained] | 265 |
| 21 | limit 20 to yr="2010 -Current" [Limit not valid in DARE; records were retained] | 189 |
| 22 | remove duplicates from 21 | 184 |

## Table S2. Electronic search strings for the SLR update

| **Embase** | | |
| --- | --- | --- |
| **#** | **Searches** | **Results** |
| 1 | Alzheimer disease/ or Alzheimer*.ti,ab. | 260959 |
| 2 | epidemiology/ or epidemiology.ti,ab,kw. | 471985 |
| 3 | incidence/ or incidence.ti,ab,kw. | 1304344 |
| 4 | prevalence/ or prevalence.ti,ab,kw. | 1184219 |
| 5 | mortality/ or life expectancy/ or mortality rate/ | 926465 |
| 6 | (mortality or mortaliti*).tw,ot. | 1255897 |
| 7 | morbidity/ or comorbidity/ | 672041 |
| 8 | (morbidity or morbiditi* or comorbidity or comorbiditi*).tw,ot. | 884015 |
| 9 | (burden of disease or disability adjusted life year or disability-adjusted life year or DALY or years of life lost or YLL or years lived with disability or YLD).ti,ab,kw. | 21500 |
| 10 | or/2-9 | 4308703 |
| 11 | 1 and 10 | 24956 |
| 12 | Clinical study/ | 156581 |
| 13 | Case control study/ | 179880 |
| 14 | Family study/ | 25359 |
| 15 | Longitudinal study/ | 163198 |
| 16 | Retrospective study/ | 1156956 |
| 17 | Prospective study/ | 724546 |
| 18 | Randomized controlled trials/ | 214102 |
| 19 | 17 not 18 | 716224 |
| 20 | Cohort analysis/ | 771723 |
| 21 | (Cohort adj (study or studies)).mp. | 373171 |
| 22 | (Case control adj (study or studies)).tw. | 148560 |
| 23 | (follow up adj (study or studies)).tw. | 67534 |
| 24 | (observational adj (study or studies)).tw. | 202708 |
| 25 | (epidemiologic$ adj (study or studies)).tw. | 113249 |
| 26 | (cross sectional adj (study or studies)).tw. | 268057 |
| 27 | (registry or register$ or survey).ti,ab. | 1228232 |
| 28 | (real world or RWE).ti,ab. | 94174 |
| 29 | Real-life.ti,ab. | 39120 |
| 30 | or/12-16,19-29 | 4259162 |
| 31 | 11 and 30 | 7325 |
| 32 | (animal$ not human$).sh,hw. | 4557601 |
| 33 | 31 not 32 | 7231 |
| 34 | limit 33 to (editorial or erratum or letter or note or patent or reports or "conference review" or "review") | 946 |
| 35 | 33 not 34 | 6285 |
| 36 | case study/ or case report.tw. | 545433 |
| 37 | 35 not 36 | 6256 |
| 38 | limit 37 to english language | 6057 |
| 39 | limit 38 to yr="2010 -Current" | 4820 |
| 40 | Conference Abstract.pt. | 4245895 |
| 41 | 39 and 40 | 1668 |
| 42 | limit 41 to yr=2015-current | 1083 |
| 43 | 41 not 42 | 585 |
| 44 | 39 not 43 | 4235 |
| 45 | remove duplicates from 44 | 4191 |
| 46 | limit 45 to yr=2020-current | 1110 |
| **MEDLINE** | | |
| # | Searches | Results |
| 1 | Alzheimer disease/ or Alzheimer*.ti,ab. | 173442 |
| 2 | epidemiology/ or epidemiology.ti,ab,kw. | 210727 |
| 3 | incidence/ or incidence.ti,ab,kw. | 929059 |
| 4 | prevalence/ or prevalence.ti,ab,kw. | 786706 |
| 5 | mortality/ or life expectancy/ or mortality rate/ | 63195 |
| 6 | (mortality or mortaliti*).tw,ot. | 856555 |
| 7 | morbidity/ or comorbidity/ | 150711 |
| 8 | (morbidity or morbiditi* or comorbidity or comorbiditi*).tw,ot. | 563235 |
| 9 | (burden of disease or disability adjusted life year or disability-adjusted life year or DALY or years of life lost or YLL or years lived with disability or YLD).ti,ab,kw. | 15203 |
| 10 | or/2-9 | 2725956 |
| 11 | 1 and 10 | 13429 |
| 12 | Epidemiologic studies/ or exp case control studies/ or exp cohort studies/ or Case control.tw. or (cohort adj (study or studies)).tw. or Cohort analy$.tw. or (Follow up adj (study or studies)).tw. or (observational adj (study or studies)).tw. or Longitudinal.tw. or Retrospective.tw. or Cross sectional.tw. or Cross-sectional studies/ or (registry or register$ or survey).ti,ab. or (real world or RWE).ti,ab. or Real-life.ti,ab. | 4026365 |
| 13 | 11 and 12 | 5221 |
| 14 | (animal$ not human$).sh,hw. | 4869793 |
| 15 | 13 not 14 | 5210 |
| 16 | limit 14 to (editorial or erratum or letter or note or patent or reports or "conference review" or "review") [Limit not valid in Ovid MEDLINE(R),Ovid MEDLINE(R) Daily Update,Ovid MEDLINE(R) PubMed not MEDLINE,Ovid MEDLINE(R) In-Process,Ovid MEDLINE(R) Publisher; records were retained] | 206013 |
| 17 | 15 not 16 | 5210 |
| 18 | case study/ or case report.tw. | 2282150 |
| 19 | 17 not 18 | 5181 |
| 20 | limit 19 to english language | 4914 |
| 21 | limit 20 to yr="2010 -Current" | 3074 |
| 22 | remove duplicates from 21 | 3067 |
| 23 | limit 22 to yr="2020 -Current" | 752 |
| **Cochrane** | | |
| # | Searches | Results |
| 1 | Alzheimer disease/ or Alzheimer*.ti,ab. | 12675 |
| 2 | epidemiology/ or epidemiology.ti,ab,kw. | 13419 |
| 3 | incidence/ or incidence.ti,ab,kw. | 131581 |
| 4 | prevalence/ or prevalence.ti,ab,kw. | 44850 |
| 5 | mortality/ or life expectancy/ or mortality rate/ | 1177 |
| 6 | (mortality or mortaliti*).tw,ot. | 95908 |
| 7 | morbidity/ or comorbidity/ | 4855 |
| 8 | (morbidity or morbiditi* or comorbidity or comorbiditi*).tw,ot. | 67316 |
| 9 | (burden of disease or disability adjusted life year or disability-adjusted life year or DALY or years of life lost or YLL or years lived with disability or YLD).ti,ab,kw. | 1379 |
| 10 | or/2-9 | 286471 |
| 11 | 1 and 10 | 1172 |
| 12 | Epidemiologic studies/ or exp case control studies/ or exp cohort studies/ or Case control.tw. or (cohort adj (study or studies)).tw. or Cohort analy$.tw. or (Follow up adj (study or studies)).tw. or (observational adj (study or studies)).tw. or Longitudinal.tw. or Retrospective.tw. or Cross sectional.tw. or Cross-sectional studies/ or (registry or register$ or survey).ti,ab. or (real world or RWE).ti,ab. or Real-life.ti,ab. | 335226 |
| 13 | 11 and 12 | 364 |
| 14 | (animal$ not human$).sh,hw. | 2210 |
| 15 | 13 not 14 | 364 |
| 16 | limit 14 to (editorial or erratum or letter or note or patent or reports or "conference review" or "review") [Limit not valid in ACP Journal Club,CDSR,CCTR,DARE,CLEED,CLHTA,CLCMR; records were retained] | 0 |
| 17 | 15 not 16 | 364 |
| 18 | case study/ or case report.tw. | 2590 |
| 19 | 17 not 18 | 363 |
| 20 | limit 19 to english language [Limit not valid in ACP Journal Club,CDSR,DARE,CLCMR; records were retained] | 290 |
| 21 | limit 20 to yr="2010 -Current" [Limit not valid in DARE; records were retained] | 214 |
| 22 | remove duplicates from 21 | 209 |
| 23 | limit 22 to yr="2020 -Current" [Limit not valid in DARE; records were retained] | 24 |

## Table S3. Prevalence and incidence estimates for MCI due to AD and AD dementia in cohorts from the general population

| **First author and year** | **Country** | **Study period** | **Data source** | **Design** | **Method of diagnosis of AD** | **Population** | **Prevalence or incidence data** |
| --- | --- | --- | --- | --- | --- | --- | --- |
| **Prevalence – population-based** | | | | | | | |
| El Tallawy et al. (2019) ([34](#_ENREF_34)) | Egypt | 2006–2012 | General population of Upper Egypt | Prospective | DSM-IV-R and CASI | N = 12,508 (≥ 50 years old) | Prevalence of AD dementia: 1% |
| Grande et al. (2020) ([39](#_ENREF_39)) | Italy | 2002–2006 | Italian Health Search Database | Retrospective | ICD-9-CM codes | N = 20,779 (1,889 with AD dementia; 18,890 controls; ≥ 60 years old) | Prevalence of AD dementia in 2016: 0.8% |
| Jia et al. (2020) ([41](#_ENREF_41)) | China | 2015–2018 | General population sample | Prospective | NIA-AA or NINCDS-ADRDA criteria | N = 46,011 (≥ 60 years old) | Age- and sex-adjusted prevalence of AD dementia: 3.9% (95% CI: 3.8–4.1) |
| Rajan, et al.  (2019) ([51](#_ENREF_51)) | USA | 1993–2012 | CHAP population-based study | Prospective | Neurologic examination, battery of 19 neurocognitive tests and NINCDS-ADRDA criteria | N = 2,794 (≥ 65 years old) | US population standardized prevalence of AD dementia: 14.5% (95% CI: 13.7–15.3)  CHAP prevalence of AD dementia: 23.5% (95% CI: 22.6–24.3) |
| Ruano et al.  (2019) ([54](#_ENREF_54)) | Portugal | 2013–2015 | EPIPorto population-based cohort | Unclear | Clinical, imaging, and laboratory data retrieved from health records, based on the DSM-V criteria | N = 730 (≥ 55 years old) | Prevalence of MCI due to AD or AD dementia: 1.8%  Prevalence of MCI due to AD: 1.4%  Prevalence of AD dementia: 0.4% |
| Vlachos et al. (2020) ([58](#_ENREF_58)) | Greece | NR | The HELIAD study | Prospective | Petersen criteria | N = 1,960  (≥ 65 years old, recruited from the general population) | Prevalence of MCI due to AD: 8.4% |
| **Prevalence – over time** | | | | | | | |
| Honda (2019) (abstract) ([40](#_ENREF_40)) | Japan | 1986–2016 | Hisayama cohort study | Prospective | Brain pathology related to AD investigated using automated morphometric analyses for quantifying tau pathology | N = 1,371 autopsies performed during study period | Prevalence of AD dementia and of tau pathology increased significantly over the study period in both men and women (statistical significance NR) |
| Sutovskyy et al.  (2018) ([57](#_ENREF_57)) | Slovakia | 2004 and 2011 | Cohort study of 10 assisted living facilities | Prospective | MMSE, CDR, DSM-IV criteria | 2004: N = 866 2011: N = 821  (92% of individuals were ≥ 65 years old) | Prevalence of AD:  2004: 24%  2011: 28% |
| Wan Suh et al. (2021) ([61](#_ENREF_61)) | South Korea | 2008–2017 | NaSDEK | Prospective | NINCDS-ADRDA criteria | 2008: N = 6,141 (≥ 65 years old) 2017: N = 2,972 (≥ 65 years old) | Age- and sex-standardized prevalence of AD  2008: 7.6%  2017: 6.8% |
| **Incidence** | | | | | | |  |
| Andreu-Reinon et al. (2020) ([22](#_ENREF_22)) | Spain | 1992–2017 | EPIC-Spain Dementia Cohort | Prospective | Medical records, verified by algorithm and neurologist review | N = 25,015 (30–70 years old, recruited from the general population) | Age-adjusted incidence of AD per 1000 person-years in individuals ≥ 65 years old, standardized to the 2013 European Standard Population: 5.47 (95% CI: 4.53–6.42) |
| Kirson et al. (2020) ([42](#_ENREF_42)) | USA | 2007–2014 | Medicare database | Retrospective | ICD-9-CM codes | 2007: 1,065,395  2008: 1,041,171  2009: 1,035,016  2010: 1,031,348  2011: 1,046,063  2012: 1,048,673  2013: 1,052,816  2014: 1,058,482  (Random sample of US Medicare beneficiaries aged ≥ 65 years) | Incidence of AD (%)  2007: 1.5  2008: 1.6  2009: 1.7  2010: 1.4  2011: 1.4  2012: 1.3  2013: 1.2  2014: 1.1 |
| Rajan, et al.  (2019) ([51](#_ENREF_51)) | USA | 1993–2012 | CHAP population-based study | Prospective | Neurologic examination, battery of 19 neurocognitive tests and NINCDS-ADRDA criteria | N = 2,794 (≥ 65 years old) | US population standardized incidence of AD dementia: 2.3% (95% CI: 1.7–2.9)  CHAP incidence of AD dementia: 3.6% (95% CI: 3.3–3.9) |
| Vlachos et al. (2021) ([59](#_ENREF_59)) | Greece | 2009–2019 | HELIAD study | Prospective | Petersen criteria | N = 955 (≥ 65 years old) | Incidence of MCI due to AD dementia per 1,000 person-years: 34.1 (95% CI: 33.9–34.3) |
| Vlachos et al. (2021) ([60](#_ENREF_60)) | Greece | 2009–2019 | HELIAD study | Prospective | DSM-IV criteria | N = 1,072 (≥ 65 years old) | Incidence of AD dementia per 1,000 person-years: 16.32 (95% CI: 16.2–16.5) |
| Yuan, et al.  (2016) ([64](#_ENREF_64)) | China | 1997–2002 | Population-based cohort study | Prospective | NINCDS-ADRDA criteria | N = 16,921 (≥ 55 years old)^a^ | Crude incidence of AD dementia per 1,000 person-years: 4.9 (95% CI: 4.3–5.7) |

^a^A total of 4,040 individuals were lost to follow-up, but this was not accounted for in the analyses.

AD, Alzheimer’s disease; CASI, Cognitive Abilities Screening Instrument; CDR, Clinical Dementia Rating; CHAP, Chicago Health and Aging Project; CI, confidence interval; DSM-IV, Diagnostic and Statistical Manual of Mental Disorders, Fourth Edition; DSM-IV-R, Diagnostic and Statistical Manual of Mental Disorders, Fourth Edition-Revised; DSM-V, Diagnostic and Statistical Manual of Mental Disorders, Fifth Edition; EPIC, European Prospective Study on Nutrition and Cancer; HELIAD, Hellenic Longitudinal Investigation of Aging and Diet; ICD, International Classification of Diseases; ICD-9-CM, International Classification of Diseases, Ninth Revision, Clinical Modification; MCI, mild cognitive impairment; MMSE, Mini-Mental State Examination; NaSDEK, Nationwide Survey on Dementia Epidemiology of Korea; NIA-AA, National Institute on Aging/Alzheimer’s Association; NINCDS-ADRDA, National Institute of Neurological and Communicative Disorders and Stroke–Alzheimer’s Disease and Related Disorder Association; NR, not reported.

## Table S4. Comorbidities reported among people with MCI due to AD or AD dementia

| **First author and year** | **Country** | **Study period** | **Data source** | **Design** | **Method of diagnosis of AD** | **Population** | **Age of patients with MCI due to AD or AD dementia, mean ± SD, years** | **Comorbidities (% of all patients at baseline)** | | |
| --- | --- | --- | --- | --- | --- | --- | --- | --- | --- | --- |
|  |  |  |  |  |  |  |  | **Hypertension** | **Diabetes** | **CVD** |
| Chen et al. (2019) ([83](#_ENREF_83)) | Taiwan | 2012–2016 | Veterans’ Home | Prospective | NIA-AA and NINCDS-ADRDA criteria | N = 84 with AD dementia (at end of study period, alive: n = 53; deceased: n = 31) | All: 86.6 ± 3.9  Alive: 85.8 ± 3.1  Deceased: 87.9 ± 4.7 | All: 60.7% Alive: 58.5%  Deceased: 64.5% | All: 17.9%^a^  Alive: 15.1%  Deceased: 22.6% | All: 21.4%  Alive: 20.8%  Deceased: 22.6%  Cerebrovascular disease:  All: 15.5%^a^  Alive: 17.0%  Deceased: 12.9% |
| Chen et al. (2020) ([82](#_ENREF_82)) | Taiwan | 2015–2016 | Dementia outpatient clinic | Prospective | Clinical examination, MMSE, and CDR | N = 80 with AD dementia | 76.0 ± 8.9 | 37.5% | 22.5% | Hyperlipidemia: 18.8% |
| de Mauleon et al. (2017) ([30](#_ENREF_30)) | France | 2007–2011 | ROSAS study | Prospective | DSM-IV-TR criteria, MMSE, and CDR | N = 184 with AD dementia | 79.5 ± 6.0 | 56.0% | 9.2% | History of or ongoing ischemic and hemorrhagic stroke: 3.8% |
| Gracner et al. (2021) ([38](#_ENREF_38)) | USA | 2011–2013 | Nursing home residents in the Minimum Data set | Retrospective | Minimum Data Set item I4200 and cognitive function score of 4 (severely impaired) | N = 12,093 with AD dementia | 65–74: 10.5%  75–84: 38.3%  85–94: 45.6%  ≥ 95: 5.6% | 71.1% | 24.3% (T2D) | Heart failure: 15.2%  Stroke:  12.5% |
| Grande et al. (2021) ([39](#_ENREF_39)) | Italy | 2002–2006 | Italian Health Search Database | Retrospective | ICD-9-CM codes | N = 1,889 with AD dementia | ≥ 60 years | In the 10-year or longer medical history before diagnosis:  48.5% | In the 10-year or longer medical history before diagnosis:  10.6% | In the 10-year or longer medical history before diagnosis  Atrial fibrillation:  0.3%  Ischemic cardiomyopathy:  5.0%  Stroke:  2.7%  Dyslipidemia: 33.5% |
| Kelaiditi et al. (2016) ([86](#_ENREF_86)) | 12 European countries | 2003–2005 | Impact of Cholinergic Treatment Use study | Prospective | NINCDS-ADRDA; MMSE | N = 1,191 with AD dementia | 76.2 ± 7.6 | 39.0% | 11.6% | Ischemic heart disease: 13.2%  Stroke: 8.0% |
| Mueller *et al*. (2018) ([90](#_ENREF_90)) | UK | 2008–2012 | South London and Maudsley NHS Foundation Trust Clinical Record  Interactive Search application | Retrospective | ICD-10^b^ | N = 2,464 with AD dementia (AChEI receipt, n = 1,261; AChEI receipt, n = 1,203) | ≥ 65 years (At dementia diagnosis: AChEI receipt: 80.8 ± 6.6; AChEI non-receipt: 83.1 ± 7.0) | NR | NR | Circulatory disease (ICD code I00–I99)  AChEI receipt: 15.4%  AChEI non-receipt: 23.9% |
| Pan et al. (2021) ([91](#_ENREF_91)) | Taiwan | 2001–  2019 | Chang Gung Research Database | Retrospective | ICD-9-CM codes, certified by neurologists based on imaging and blood and cognitive tests | N = 3,846 with AD dementia | 77.8 ± 6.2 | 52.9% | 24.2% (T2D) | Hyperlipidemia: 29.7% |
| Rhodius-Meester *et al*. (2018) ([92](#_ENREF_92)) | Netherlands | 2000–2014 | Amsterdam Dementia Cohort | Prospective | NINCDS-ADRDA and NIA-AA criteria | N = 616 with AD dementia (Alive after mean ± SD follow-up of 4.9 ± 2.0 years, n = 403; deceased, n = 213) | Alive: 66 ± 7  Died: 69 ± 9 | History of hypertension and/or use of antihypertensive drugs)  Alive: 31.5%  Deceased: 36.2% | History of Diabetes  and/or use of antidiabetic drugs  Alive: 7.7%  Deceased: 7.0% | History of coronary  heart disease, heart failure, heart disease, peripheral  vascular disease, stroke, and/or transient ischemic  attack  Alive: 17.6%  Deceased: 24.9% |
| Staekenborg et al. (2016) ([94](#_ENREF_94)) | Netherlands | 2000–2013 | Amsterdam Dementia Cohort | Retrospective | NIA-AA clinical criteria | N = 685 with AD dementia (n = 55 with rapid mortality [death within ≤ 2 years after diagnosis]; n = 630 with non-rapid mortality) | NR | Rapid mortality: 38%  Non-rapid mortality: NR | Rapid mortality: 5.5%  Non-rapid mortality: NR | Cardiac history^c^ Rapid mortality: 13%  Non-rapid mortality: NR  TIA/stroke  Rapid mortality: 5.5%  Non-rapid mortality: NR |
| Vu et al. (2020) ([95](#_ENREF_95)) | Finland | 2005–2011 | The register–based MEDALZ cohort | Retrospective | NINCDS-ADRDA and DSM-IV criteria, including CT or MRI | N = 65,423 with AD dementia | 79.9 ± 7.1 | At the time of AD diagnosis:  41.5% | At the time of AD diagnosis:  18.1% | At the time of AD diagnosis  Atrial fibrillation: 15.4%  Coronary artery disease:  29.0%  Stroke:  9.8% |
| Xu et al. (2021) ([62](#_ENREF_62)) | Sweden | 2007–2017 | Swedish Dementia Registry | Retrospective | MMSE | N = 10,129 with AD dementia (AChEI nonusers: n = 5,826; AChEI users: n = 11,652) | AChEI nonuser: 81.1 ± 6.7  AChEI user: 81.2 ± 6.1 | AChEI non-users: 73.9%  AChEI users: 73.8% | AChEI non-users: 15.7%  AChEI users: 15.6% | Myocardial infarction  AChEI non-users: 10.0%  AChEI users: 9.8% |
| Yeh et al. (2020) ([96](#_ENREF_96)) | Taiwan | 2000–2012 | NHIRD; National Mortality Registry | Retrospective | AChEIs prescription required  (MMSE or CDR; CT and/or MRI) | N = 21,615 with AD dementia  (Early-onset, n = 1,257; late-onset, n = 20,358) | Early-onset AD (mean ± SE): 60.6 ± 4.4  Late-onset AD: 77.5 ± 6.4 | Early-onset AD: 30.2%  Late-onset AD: 51.3% | Early-onset AD: 20.7%  Late-onset AD: 23.5% | CVD  Early-onset AD: 12.2%  Late-onset AD: 19.7%  Stroke:  Early-onset AD: 10.8%  Late-onset AD: 13.7% |

^a^Calculated from values for alive and deceased individuals. ^b^Excludes individuals who died within 6 months of first AD diagnosis. ^c^History of angina/myocardial infarction/heart valve disorder/cardiac rhythm disorders.

AChEI, acetylcholinesterase inhibitor; AD, Alzheimer’s disease; CDR, Clinical Dementia Rating; CT, computed tomography; CVD, cardiovascular disease; DSM-IV, Diagnostic and Statistical Manual of Mental Disorders (Fourth Edition); DSM-IV-TR, Diagnostic and Statistical Manual of Mental Disorders (Fourth Edition) – Text Revision; ICD-9-CM, International Classification of Diseases, Ninth Revision, Clinical Modification; ICD-10, International Classification of Diseases, Tenth Revision; MCI, mild cognitive impairment; MEDALZ, Medication Use and Alzheimer’s disease; MMSE, Mini-Mental State Examination; MRI, magnetic resonance imaging; NHIRD, National Health Insurance Research Database; NIA-AA, National Institute on Aging/Alzheimer’s Association; NINCDS-ADRDA, National Institute of Neurological and Communicative Disorders and Stroke–Alzheimer’s Disease and Related Disorder Association; NR, not reported; ROSAS, Research Of biomarkers in Alzheimer’s diseaSe; SE, standard deviation; SE, standard error; TIA, transient ischemic attack; T2D, type 2 diabetes

## Table S5. Comorbidities among people with AD dementia and controls

| **First author and year** | **Country**  **Data source**  **Study period** | **Design** | **Method of diagnosis of AD** | **Population** | **Population age, mean ± SD, years** | **Comorbidities** |
| --- | --- | --- | --- | --- | --- | --- |
| Andersen et al. (2011) ([65](#_ENREF_65)) | Norway  The Dementia Study  2006–2008 | Prospective | ICD-10 criteria and DSM-IV-TR. Diagnostic discrepancies were solved by consensus advised by NINCDS-ADRDA criteria for probable AD | N = 387  (187 with AD dementia; 200 controls) | AD dementia: 80.9 ± 7.0^a^  Controls: 72.5 ± 5.5 | CVD:^b^ n (%)  AD dementia: 139 (74.3)  Controls: 114 (57.0)  Unadjusted p < 0.001  Adjusted for age and sex p = 0.14  MI, n (%)  AD dementia: 27 (14.4)  Controls: 24 (12.0)  Unadjusted p < 0.52  Adjusted for age and sex p = 0.26  Hypertension, n (%)  AD dementia: 102 (54.5)  Controls: 82 (41.0)  Unadjusted p = 0.008  Adjusted for age and sex p = 0.14  Stroke, n (%)  AD dementia: 33 (17.6)  Controls: 11 (5.5)  Unadjusted p < 0.001  Adjusted for age and sex p = 0.82  Diabetes, n (%)  AD dementia: 21 (11.2)  Controls: 17 (8.5)  Unadjusted p = 0.37  Adjusted for age and sex p = 0.15  Chronic obstructive bronchitis, n (%)  AD dementia: 19 (10.2)  Controls: 10 (5.0)  Unadjusted p = 0.054  Adjusted for age and sex p = 0.003 |
| de Lima et al. (2020) ([66](#_ENREF_66)) | Brazil  Center of Alzheimer Disease of the Psychiatry Institute of the Federal University of Rio de Janeiro  NR | Prospective | DSM-IV and Petersen criteria for dementia and MCI | 212 (54 with AD dementia, 26 with MCI, 30 with major depressive disorder; 102 controls) | AD dementia: 79.6 ± 7.9^a^  MCI: 76.7 ± 6.3  Controls: 72.7 ± 7.9 years | Hypertension, %  AD dementia: 51.9  MCI: 53.8  Controls: 56.9  OR (AD dementia versus controls): 0.81 (95% CI: 0.42–1.58); p = 0.613  Diabetes, %  AD dementia: 25.9  MCI: 7.7  Controls: 16.7  OR (AD dementia versus controls): 1.75 (95% CI: 0.78–3.89); p = 0.206)  Dyslipidemia, %  AD dementia: 38.9  MCI: 26.9  Controls: 18.6  OR (AD dementia versus controls): 2.78 (95% CI: 1.32–5.82); p = 0.007 |
| Grande et al. (2020) ([39](#_ENREF_39)) | Italy  Italian Health Search Database  2002–2006 | Retrospective | ICD-9-CM codes | 20,779 (1,889 with AD dementia; 18,890 controls) | AD dementia: ≥ 60 years  Controls: NR | Hypertension, n (%)  AD dementia: 916 (48.5)  Controls: 9,462 (50.1)  OR: 0.93 (95% CI: 0.84–1.03); p = 0.16  Diabetes, n (%)  AD dementia: 200 (10.6)  Controls: 1,743 (9.2)  OR: 1.17 (95% CI: 1.00–1.37); p = 0.05  Stroke, n (%)  AD dementia: 50 (2.7)  Controls: 501 (2.7)  OR: 1.00 (95% CI: 0.74–1.34); p = 0.99  Atrial fibrillation, n (%)  AD dementia: 6 (0.3)  Controls: 104 (0.6)  OR: 0.58 (95% CI: 0.25–1.31); p = 0.1  Ischemic cardiomyopathy, n (%)  AD dementia: 94 (5.0)  Controls: 1,059 (5.6)  OR: 0.88 (95% CI: 0.71–1.09); p = 0.25  Kidney disease, n (%)  AD dementia: 98 (5.2)  Controls: 1,020 (5.4)  OR: 0.96 (95% CI: 0.77–1.19); p = 0.69  Dyslipidemia, n (%)  AD dementia: 632 (33.5)  Controls: 6,292 (33.3)  OR: 1.01 (95% CI: 0.91–1.12); p = 0.89  Depression, n (%)  AD dementia: 374 (19.8)  Controls: 2,029 (10.7)  OR: 2.07 (95% CI: 1.83–2.35); p < 0.001 |
| Ilmaniemi *et al*. (2019) ([67](#_ENREF_67)) | Finland  The register–based MEDALZ cohort  2005–2015 | Retrospective | NINCDS-ADRDA and DSM-IV criteria; symptoms consistent with mild or moderate AD dementia^c^ | 134,344 (67,172 with AD dementia; 67,172 controls) | AD dementia: 80.0 (95% CI: 79.9–80.0)  Controls: 80.0 (95% CI: 79.9–80.0) | CVD, n (%)  AD dementia: 33,923 (50.5)  Controls: 33,021 (49.2)  p < 0.001  Stroke, n (%)  AD dementia: 6,255 (9.3)  Controls: 5,359 (8.0)  p < 0.001  Diabetes, n (%)  AD dementia: 8,931 (13.3)  Controls: 7,706 (11.5)  p < 0.001  Asthma or COPD, n (%)  AD dementia: 5,826 (8.7)  Controls: 6,089 (9.1)  p = 0.012 |
| Kuyumcu et al. (2012) ([68](#_ENREF_68)) | Turkey  Outpatient geriatric clinic  NR | Prospective | DSM-IV and NINCDS-ADRDA criteria after cognitive assessment (MMSE, clock drawing tests) and neuroimaging (MRI) had been performed | 2,053 (193 with AD dementia; 1,860 controls) | AD dementia: 74.4 ± 7.2  Controls 71.9 ± 5.9 | Hypertension, n (%)  AD dementia: 117 (60.6)  Controls: 1,311 (70.5)  p = 0.005  OR: 0.65 (95% CI: 0.46–0.92); p = 0.014  CAD, n (%)  AD dementia: 64 (33.2)  Controls: 497 (26.7)  p = 0.056  CHF, n (%)  AD dementia: 16 (8.3)  Controls: 133 (7.2)  p = 0.561  Diabetes, n (%)  AD dementia: 35 (18.1)  Controls: 429 (23.1)  p = 0.119 |
| Lanyau-Dominguez et al. (2020) ([69](#_ENREF_69)) | Cuba  Aging and Alzheimer Study (Medical University of Havana’s Alzheimer Research Group)  NR | Prospective | International 10/66 dementia diagnostic criteria and DSM-IV were used to diagnose dementia. MCI was diagnosed using Petersen's criteria | 424 (43 with AD dementia, 131 with MCI, 250 controls) | AD dementia: 82.8 years;^d^  MCI: 79.5 years^d^  Controls: NR | Hypertension, %  AD dementia: 72.1  MCI: 74.8  Controls: 73.2  Glucose metabolism disorder, %  AD dementia: 58.5  MCI: 55.4  Controls: 49.8 |
| Lee et al. (2016) (abstract) ([70](#_ENREF_70)) | Taiwan  National Health Insurance Research database  NR | Retrospective | NR | 3,908 (977 with AD dementia; 2,931 controls) | NR | Risk of urge incontinence for AD dementia vs matched controls, HR: 1.34 (95% CI: 1.12–1.61)  The cumulative incidence ratio of urge incontinence event between AD dementia cohort and matched controls cohort was statistically significant (p = 0.006) |
| Lee et al. (2017) ([71](#_ENREF_71)) | Taiwan  National Health Insurance Research database  2000–2010 | Retrospective | DSM-IV criteria in addition to brain neuroimaging and neuropsychological assessments required for the use of AChEIs in Taiwan | 3,732 (933 with AD dementia; 2,799 controls) | AD dementia: 75.2 ± 7.7  Controls: 74.9 ± 7.8 | Risk of urge incontinence for AD dementia vs matched controls, HR: 1.54 (95% CI: 1.13–2.09), after adjusting for age, sex, diabetes, hypertension chronic kidney disease, and urinary tract infection  Hypertension, n (%)  AD dementia: 597 (64.0)  Controls: 1795 (64.1)  p = 0.937  Diabetes, n (%)  AD dementia: 274 (29.4)  Controls: 820 (29.3)  p = 0.967  Chronic kidney disease, n (%)  AD dementia: 50 (5.4)  Controls: 126 (4.5)  p = 0.285  Urinary tract infection, n (%)  AD dementia: 282 (30.2)  Controls: 837 (29.9)  p = 0.853 |
| Lee et al. (2019a) (abstract) ([72](#_ENREF_72)) | Korea  National Health Insurance Service-National Elderly cohort (NHIS-elderly) database  NR | Prospective | KCD codes G30 and F00 | NR | NR | Risk of epilepsy for AD dementia vs controls, HR: 2.77 (95% CI: 2.52–3.06) |
| Lee et al. (2019b) (abstract) ([73](#_ENREF_73)) | Korea  National Health Insurance Service -Senior cohort  2002–2013 | Retrospective | NR | 22,537 (3,524 with AD dementia;^e^ 19,013 controls) | NR | Risk of stroke for AD dementia vs matched controls, HR: 2.87 (95% CI: 2.71–3.04), after adjusting for other risk factors (not reported) |
| Lyou et al. (2018) ([74](#_ENREF_74)) | Korea  National Health Insurance Service-National Elderly cohort database  2004–2013 | Retrospective | KCD codes G30 and F00 related to AD dementia as claim codes in principal and minor diagnoses, excluding cases with F01 as claim code known to be related to vascular dementia | 24,229 (4,516 with AD dementia; 19,713 controls) | NR | Diabetes, n (%)  AD dementia: 2,028 (44.9)  Controls: 5,928 (30.1)  p < 0.0001  Hypertension, n (%)  AD dementia: 3,238 (71.7)  Controls: 10,845 (55.0)  p < 0.0001  CKD, n (%)  AD dementia: 140 (3.1)  Controls: 266 (1.4)  p < 0.0001  Dyslipidemia, n (%)  AD dementia: 1,720 (38.1)  Controls: 4,444 (22.5)  p < 0.0001  Seizure, n (%)  AD dementia: 631 (14.0)  Controls: 1,193 (6.1)  p < 0.0001  Tumor, n (%)  AD dementia: 18 (0.4)  Controls: 35 (0.2)  p = 0.004 |
| Sannemann et al. (2020) ([75](#_ENREF_75)) | Germany  DELCODE study  NR | Prospective | Clinical examination and neuropsychological testing (criteria NR) | 687 (77 with AD dementia, 115 with MCI, 209 controls; biomarker data to confirm diagnosis available for 39 with AD dementia, 74 with MCI and 76 controls) | AD dementia: 73.7 ± 6.7^a^  MCI: 72.1 ± 5.3^a^  Controls: 68.8 ± 5.3 | Number of reported NPI-Q Items, mean ± SD:  AD dementia (n = 77): 2.6 ± 2.5  MCI (data for n = 113): 2.2 ± 2.0  Controls (data for n = 203): 0.4 ± 0.8  NPS ≥ 1, n (%):  AD dementia: 56 (72.7)  MCI: 83 (73.5)  Controls: 53 (26.1)  NPS ≥ 2, n (%):  AD dementia: 48 (62.3)  MCI: 68 (60.2)  Controls: 23 (11.3)  GDS-15 total score, mean ± SD:  AD dementia (data for n = 75): 2.2 ± 1.8  MCI (n = 109): 2.1 ± 1.9  Controls (n = 204): 0.7 ± 1.3  Subthreshold symptoms of depression (GDS-15 score ≥ 1), n (%):  AD dementia: 62 (82.7)  MCI: 91 (83.5)  Controls: 74 (36.3)  GAI-SF total score, mean ± SD:  AD dementia (data for n = 77): 1.1 ± 1.2  MCI (n = 115): 1.0 ± 1.1  Controls (n = 209): 0.7 ± 0.8  Subthreshold symptoms of anxiety (GAI-SF score ≥ 1), n (%):  AD dementia: 46 (59.7)  MCI: 65 (56.5)  Controls: 103 (49.3) |
| Tolppanen et al. (2013a) ([76](#_ENREF_76)) | Finland  MEDALZ-2005  2002–2009 | Retrospective (2002–2005) and prospective (2006–2009) | NINCDS-ADRDA and DSM-IV criteria^c^ | 50,650 (27,123 with AD dementia; 23,527 controls) | AD dementia: 79.5 ± 6.9  Controls: 79.5 ± 6.9 | Risk of incident ischemic heart disease for AD dementia vs controls  Model 1 (unadjusted):  HR 0.93 (95% CI: 0.85–1.01); p = 0.09  Model 2 (adjusted for stroke and other comorbidities):  HR 0.94 (95% CI: 0.86–1.02); p = 0.14  Model 3 (adjusted for stroke and other comorbidities, number of cardiovascular medicines, diabetes medication, and antithrombotic medication at baseline):  HR 0.95 (95% CI: 0.87–1.04); p = 0.30  History of stroke, n (%)  AD dementia: 2,502 (9.9)  Controls: 2,529 (10.0)  p = 0.69  Diabetes, n (%)  AD dementia: 3,963 (15.7)  Controls: 3,727 (14.7)  p = 0.003 |
| Tolppanen et al. (2013b) ([77](#_ENREF_77)) | Finland  MEDALZ-2005  2002–2009 | Retrospective (2002–2005) and prospective (2006–2009) | NINCDS-ADRDA and DSM-IV criteria^c^ | 51,972 (27,789 with AD dementia; 25,183 controls) | 79.9 ± 6.8 (entire cohort; NR for AD dementia) | Prevalence of previous hip fractures  AD dementia: 4.7%  Matched controls: 2.4%  Incident hip fractures during 2006–2009: n = 2,861 people (70.5% with AD dementia; 29.5% without AD dementia)  Risk of incident hip fractures for AD dementia vs no AD dementia  Model 1 (crude analysis): HR: 2.70 (95% CI: 2.47–2.96)  Model 2 (adjusted for comorbidities):^f^  HR 2.74 (95% CI: 2.48–3.03) |
| Tolppanen et al. (2013c) ([78](#_ENREF_78)) | Finland  NR  2006–2009 | Prospective | NINCDS-ADRDA and DSM-IV criteria^c^ | 50,808 (27,170 with AD dementia; 23,638 controls) | NR | Risk of any stroke for AD dementia vs controls  Model 1: HR: 1.01 (95% CI: 0.94–1.09)  Model 2 (adjusted for cancer, diabetes, and CVDs): HR: 1.02 (95% CI: 0.94–1.10).  Risk of hemorrhagic stroke for AD dementia vs controls  Model 1: HR: 1.34 (95% CI: 1.12–1.60)  Model 2: HR: 1.34 (95% CI: 1.12–1.61)  Risk of ischemic stroke for AD dementia vs controls  Model 1: HR: 0.93 (95% CI: 0.86–1.01)  Model 2: HR: 0.94 (95% CI: 0.86–1.03) |
| Tolppanen et al. (2016) ([79](#_ENREF_79)) | Finland  MEDALZ  2005–2012 | Retrospective | NINCDS-ADRDA and DSM-IV criteria^c^ | 134,144 (67,072 with AD dementia; 67,072 controls) | AD dementia: mean 79.9 (95% CI: 79.8–79.9) years  Controls: 79.9 (95% CI: 79.8–79.9) years | Hip fracture incidence:  AD dementia: 2.23/100 person-years (n = 5,264 hip fractures)  Controls: 0.98/100 person-years (n = 2,643 hip fractures)  Risk of hip fracture during follow-up for  AD dementia vs controls, HR: 2.35 (95% CI: 2.24–2.46), adjusted for socioeconomic position  CVD, n (%)  AD dementia: 34,046 (50.8)  Controls: 32,841 (49.0)  p < 0.001  Stroke, n (%)  AD dementia: 6,356 (9.5)  Controls: 5,232 (7.8)  p < 0.001  Diabetes, n (%)  AD dementia: 8,969 (13.4)  Controls: 7,527 (11.2)  p < 0.001  Hip replacement, n (%)  AD dementia: 2,219 (3.3)  Controls: 1,001 (1.5)  p < 0.001  Knee replacement, n (%)  AD dementia: 105 (0.2)  Controls: 67 (0.1)  p = 0.004  Any mental or behavioural disorder, n (%)  AD dementia: 15,365 (22.9)  Controls: 5,917 (8.8)  p < 0.001  Asthma/COPD, n (%)  AD dementia: 5,846 (8.7)  Controls: 6,011 (9.0)  p = 0.11  Cancer treatment, n (%)  AD dementia: 792 (1.2)  Controls: 855 (1.3)  p = 0.12 |
| Vu et al. (2020) ([95](#_ENREF_95)) | Finland  The register–based MEDALZ cohort  2005–2011 | Retrospective | NINCDS-ADRDA and DSM-IV criteria, including CT or MRI | 134,741 (65,423 with AD dementia; 69,318 controls) | 79.9 ± 7.1  Controls: 79.9 ± 7.08 | Hypertension, n (%)  AD dementia: 27,159 (41.5)  Non-AD: 28,033 (40.4); p < 0.00001  Stroke, n (%)  AD dementia: 6,389 (9.8)  Non-AD: 5,567 (8.0); p < 0.00001  CAD, n (%)  AD dementia: 18,997 (29.0)  Non-AD: 18,311 (26.4); p < 0.00001  Atrial fibrillation, n (%)  AD dementia: 10,061 (15.4)  Non-AD 8,921 (12.9); p < 0.00001  Chronic heart failure, n (%)  AD dementia: 8,698 (13.3)  Non-AD: 8,185 (11.8); p < 0.00001  Diabetes, n (%)  AD dementia: 11,807 (18.1)  Non-AD: 10,482 (15.1); p < 0.00001 |
| Wang et al. (2018) ([80](#_ENREF_80)) | Taiwan  National Health Insurance program  2001–2011 | Retrospective | ICD-9 to identify patients with dementia codes. Incident cases were further selected by the approved medication reimbursement for AChEIs (rivastigmine, donepezil, galantamine) or memantine | 7,854 (2,618 with AD dementia; 5,236 controls) | AD dementia: 76.1 ± 6.5  Controls: 76.1 ± 6.5 | Hypertension, n (%)  AD dementia: 1,443 (55.1)  Controls: 2,705 (51.7)  AOR 0.94 (0.82, 1.09); p = 0.284  Osteoarthritis, n (%)  AD dementia: 1,001 (38.2)  Controls: 1,677 (32.0)  AOR 1.09 (0.95, 1.26); p = 0.111  Depression, n (%)  AD dementia: 846 (32.3)  Controls: 719 (13.7)  AOR 2.92 (2.48, 3.44); p < 0.001  Diabetes, n (%):  AD dementia: 672 (25.7)  Controls: 1,135 (21.7)  AOR 1.19 (1.01, 1.39); p = 0.005  CVD, n (%)  AD dementia: 597 (22.8)  Controls: 716 (13.7)  AOR 1.65 (1.40, 1.96); p < 0.001 |
| Yazar and Yazar (2019) ([81](#_ENREF_81)) | Turkey  Ordu University Education and Research Hospital and Ordu State hospital neurology clinics  NR | Prospective | NINCDS-ADRDA criteria | 406 (127 with AD dementia; 159 controls)  CDR stage 1: n = 43  CDR stage 2: n = 41  CDR stage 3: n = 43 | AD dementia: women: 75.8 ± 6.3; men: 75.7 ± 6.3^g^  Controls (age 70–80 years): women: 75.0 ± 4.6; men: 74.2 ± 4.8 | 72.9% of patients with severe sarcopenia were in the AD dementia group  Overall, 65.7% of patients with severe sarcopenia were in the CDR stage 3 group  Overall, 53.8% of patients with dynapenia were in the AD dementia group. The prevalence of dynapenia in women was the same in both the control 2 (age 70–80 years) and AD dementia groups. For men, the prevalence of dynapenia was higher in the AD dementia group compared with the control group |

^a^Significantly older than the cognitively-healthy group, p < 0.001); ^b^Participants experiencing one or more of congestive heart failure, MI, angina pectoris, or atrial fibrillation; ^c^People with mild or moderate AD dementia are entitled to reimbursement for anti-dementia medication in Finland, but the reimbursement is not withdrawn if the patient develops severe AD dementia; therefore the study sample included persons with all stages of AD dementia; ^d^Standard deviation not reported; ^e^Or 23,803 (4,790 with AD dementia), unclear in abstract; ^f^Adjusted for CVDs, cancer, diabetes, pernicious anemia, and other disturbances in absorption of vitamin B12, Parkinson’s disease, epilepsy, glaucoma, and rheumatoid arthritis. ^g^NR for the cohort overall.

AChEI, acetylcholinesterase inhibitor; AD, Alzheimer’s disease; ADRD, AD or related dementias; AOR, adjusted odds ratio; BMI, body mass index; CDR, Clinical Dementia Rating; CAD, coronary artery disease; CHF, congestive heart failure; CKD, chronic kidney disease; COPD, chronic obstructive pulmonary disease; CVD, cardiovascular disease; DELCODE, DZNE Longitudinal Cognitive Impairment and Dementia; DSM-IV, Diagnostic and Statistical Manual of Mental Disorders, Fourth Edition; DSM-IV-TR, DSM-IV Text Revision; DZNE, Deutsches Zentrum für Neuro-degenerative Erkrankungen [German Center for Neurodegenerative Diseases]; GAI-SF, Geriatric Anxiety Inventory-Short Form; GDS-15, Geriatric Depression Scale 15-item version; HR, hazard ratio; ICD-9, International Classification of Diseases, Ninth Revision; ICD-10, International Classification of Diseases, Tenth Revision; KCD, Korean Standard Classification of Disease; MCI, mild cognitive impairment; MEDALZ, Medication Use and Alzheimer’s disease cohort; MI, myocardial infarction; MMSE, Mini-Mental State Examination; MRI, magnetic resonance imaging; NINCDS-ADRDA, National Institute of Neurological and Communicative Disorders and Stroke–Alzheimer’s Disease and Related Disorders Association; NPI-Q, Neuropsychiatric Inventory Questionnaire; NPS, neuropsychiatric symptoms; NR, not reported; OR, odds ratio; SD, standard deviation
